# All-trans retinoic acid pretreatment of mesenchymal stem cells enhances the therapeutic effect on acute kidney injury

**DOI:** 10.1186/s12964-024-01671-1

**Published:** 2024-05-27

**Authors:** Yifan Zhang, Xiaochen Wang, Yuwei Ji, Haijuan Hong, Xiaodong Geng, Keying Zhang, Zhangning Fu, Guangyan Cai, Xiangmei Chen, Ping Li, Quan Hong

**Affiliations:** 1 Medical School of Chinese PLA, Beijing, 100853 China; 2 Department of Nephrology, First Medical Center of Chinese PLA General Hospital, National Key Laboratory of Kidney Diseases, Beijing Key Laboratory of Kidney Diseases Research, National Clinical Research Center for Kidney Diseases, No.28 Fuxing Road, Beijing, 100853 China; 3 Songjiang District Central Hospital, Shanghai, China; 4 Health Care Office of the Service Bureau of Agency for Offices Administration of the Central Military Commission, Beijing, China

**Keywords:** All-trans retinoic acid, Mesenchymal stem cells, Acute kidney injury, Hyaluronic acid

## Abstract

A promising new therapy option for acute kidney injury (AKI) is mesenchymal stem cells (MSCs). However, there are several limitations to the use of MSCs, such as low rates of survival, limited homing capacity, and unclear differentiation. In search of better therapeutic strategies, we explored all-trans retinoic acid (ATRA) pretreatment of MSCs to observe whether it could improve the therapeutic efficacy of AKI. We established a renal ischemia/reperfusion injury model and treated mice with ATRA-pretreated MSCs via tail vein injection. We found that AKI mice treated with ATRA-MSCs significantly improved renal function compared with DMSO-MSCs treatment. RNA sequencing screened that hyaluronic acid (HA) production from MSCs promoted by ATRA. Further validation by chromatin immunoprecipitation experiments verified that retinoic acid receptor RARα/RXRγ was a potential transcription factor for hyaluronic acid synthase 2. Additionally, an in vitro hypoxia/reoxygenation model was established using human proximal tubular epithelial cells (HK-2). After co-culturing HK-2 cells with ATRA-pretreated MSCs, we observed that HA binds to cluster determinant 44 (CD44) and activates the PI3K/AKT pathway, which enhances the anti-inflammatory, anti-apoptotic, and proliferative repair effects of MSCs in AKI. Inhibition of the HA/CD44 axis effectively reverses the renal repair effect of ATRA-pretreated MSCs. Taken together, our study suggests that ATRA pretreatment promotes HA production by MSCs and activates the PI3K/AKT pathway in renal tubular epithelial cells, thereby enhancing the efficacy of MSCs against AKI.

**Supplementary Information:**

The online version contains supplementary material available at 10.1186/s12964-024-01671-1.

## Introduction

Acute kidney injury (AKI) is a clinical syndrome characterized by a sudden decline in kidney function that can be life-threatening in severe cases. It is a multi-factorial syndrome that can be caused by various factors such as ischemia, nephrotoxicity, and sepsis. The high incidence and poor prognosis of AKI places a heavy burden on healthcare systems worldwide and also leads to prolonged hospitalization and increased healthcare costs. Early recognition, timely management, and prevention of AKI are essential to improve patient prognosis [1, 2]. There is still no effective therapy for AKI other than supportive care and renal replacement therapy [3]. Therefore, it is imperative to create novel, secure, and efficient treatments to lower the death rate from AKI and prevent it from leading to chronic renal disease.

Mesenchymal stem cells (MSCs) therapy holds broad promise in treating AKI [4, 5]. MSCs are characterized by high proliferation, multidirectional differentiation potentials, low immunogenicity, low tumorigenicity, stable genetic background, easy availability, and the avoidance of the ethical controversy of embryonic stem cells [6]. However, low survival rates, low homing rates and ambiguous differentiation limit the therapeutic efficacy of MSCs. To overcome these limitations, various strategies have emerged to enhance the efficacy of stem cells, including drug pretreatment, gene modification, and biomaterial encapsulation [7, 8]. Previous studies have shown that pharmacological enhancement of cellular bioactivity and improvement of the microenvironment for cell growth before transplantation is an effective pretreatment strategy for cellular therapy that can greatly benefit tissue repair [9]. Among the different strategies to modulate MSCs function, pretreatment with small molecule compounds has the benefit of convenience and safety by varying their working concentration, duration, and composition [10].

A physiologically active form of vitamin A, all-trans retinoic acid (ATRA), is essential for several physiological functions, such as immune modulation, cell differentiation, and embryonic development [11]. Clinically, ATRA is frequently used to treat various skin conditions such as psoriasis, acne, and ichthyosis [12]. In addition, ATRA was found to have an important differentiating effect on blood cells and dramatically alleviates acute promyelocytic leukemia, transforming it from one of the most aggressive types of leukemia to a curable type of leukemia [13]. Studies have shown that pretreatment with ATRA before MSCs transplantation can reduce inflammation and apoptosis, activate autophagy, and promote angiogenesis [14, 15].

In this study, we observed the impact of ATRA pretreatment of MSCs on AKI. We found that ATRA enhanced the renal repair effect of MSCs in AKI. This pretreatment strategy based on stem cell therapy improves its paracrine function and fully utilizes the renal repair of AKI by MSCs.

## Methods

### Animal model

Male C57BL/6 mice (6–8 weeks old) were purchased from the Animal Center of Academy of Chinese PLA General Hospital. The mice were randomly divided into 4 groups of 6 mice each. We established a unilateral ischemia/reperfusion injury (IRI) model by clamping the left renal artery for 30 min, followed by contralateral nephrectomy. Except for left renal artery clamping, the sham group experienced identical surgical procedures as the intervention group. The MSCs in the DMSO-MSCs group received 0.1% dimethyl sulfoxide (DMSO) treatment, serving as the reagent control group. In the DMSO-MSCs group, the mice were injected with DMSO-pretreated MSCs (1 × 10^6^ per mouse). Similarly, mice in the ATRA-MSCs group were injected with ATRA-pretreated MSCs (1 × 10^6^ per mouse). The IRI group was injected with equal volumes of phosphate-buffered saline (PBS). In the early stage of IRI, the renal inflammatory response is mild, which favors the survival of MSCs in renal tissues. In addition, increased expression of homing adhesion molecules promotes the integration of MSCs into the injured renal tissue [16]. Therefore, all mice completed tail vein injections within 1 h after ischemia. The tail vein was injected at a constant rate to reduce vein wall injury. Mice were sacrificed 3 d after surgery, and samples of kidney tissue and blood were collected. Blood samples are centrifuged at 3000 rpm for 10 min at 4 °C to isolate the serum. Blood urea nitrogen (BUN) and serum creatinine (SCr) levels were measured using a biochemical automatic analyzer (Cobas 8000, Roche Products Ltd. Basel, Switzerland).

### Glomerular Filtration Rate (GFR) measurements

GFR measurements were conducted in conscious mice using the transdermal GFR technology (MediBeacon GmBH, Mannheim, Germany). The dorsal fur of the mice was shaved off before the experiment using a shaver and depilatory cream. Mice were anesthetized using 2% isoflurane, and the MediBeacon transdermal Mini GFR monitor was attached to the depilated skin using a double-sided patch and medical tape. Fluorescein isothiocyanate (FITC)-sinistrin (70 mg/kg body weight) was injected into each mouse via the tail vein after collecting background signal for 5 min. The fluorescence signal in the skin was detected for 2 h, during which time the animals were kept free in cages. Data were analyzed using MB Studio software (MediBeacon GmBH, Mannheim, Germany).

### Histological analysis

Kidney tissues were fixed in formalin, then embedded in paraffin and sectioned to 3–5 μm in thickness. These sections were subsequently stained with Periodic Acid-Schiff (PAS). The acute tubular necrosis (ATN) score was determined by blinded histological assessments that included tubular necrosis grading, cast development, tubular dilatation, and loss of brush boundary. Ten non-overlapping fields were randomly selected and assigned scores in the following manner: 0, none; 1, ≤ 10%; 2, 11–25%; 3, 26–45%; 4, 46–75%; and 5, > 76%.

Immunohistochemistry and immunofluorescence were performed as previously described [17]. The following primary antibodies are used: KIM-1 (1:200, AF1817, R&D, USA), PCNA (1:200, ab92552, Abcam, UK), CD44 (1:200, AG1491, Biyuntian, China). We detected apoptotic cells using terminal deoxynucleotidyl transferase-mediated dUTP nick end-labeling (TUNEL) staining following the manufacturer’s protocol (KGA1408-50, KGI Biotechnology, Nanjing, China).

### Cell culture and treatment

Human umbilical cord mesenchymal stem cells (hUC-MSCs) were acquired from the Beijing Zhongyuan Company (Beijing, China). Human proximal tubular epithelial cells (HK-2) were purchased from the American Type Culture Collection (ATCC, Manassas, VA, USA). MSCs were cultured at 37 °C under 5% CO_2_ and 90% humidity, and the medium was changed every 2 d. MSCs were used at passage 6–8 for all the experiments.

Cell viability was evaluated using the Cell Counting Kit-8 (CCK-8) (CK-04, Dojindo, Kumamoto, Japan). MSCs were incubated with various concentrations of ATRA solution (concentration: 0, 0.1, 1, 1, 10, 100 µM). Control cells were cultured with a medium containing 0.1% DMSO. Wells containing the medium without cells were used as the blanks. The plates were incubated for 12, 24, and 48 h.

The hypoxia/reoxygenation (H/R) model was established using HK-2 cells. MSCs and HK-2 cells were co-cultured at a 1:3 ratio in a Transwell system. HK-2 cells were seeded in 6-well plates, while MSCs were seeded in 0.4 μm transwell chambers. HK-2 cells were cultured in glucose-free and serum-free medium under hypoxic conditions (1% O_2_, 94% N_2_ and 5% CO_2_) for 24 h, and then 24 h of co-culture with MSCs. Antibody neutralization experiments refer to previous research [18, 19]. To block HA interactions to CD44, anti-CD44 neutralizing antibody (10 µg/mL, 9400-01, Southern Biotech, USA)) was added to HK-2 cells culture medium before hypoxia treatment.

For siRNA knockdown, negative control-siRNA and hyaluronic acid synthase 2 (HAS2)-siRNA were purchased from Jima Biological Company (Suzhou, Jiangsu, China). Cell transfection was performed using Lipofectamine 3000 reagent (Invitrogen, Grand Island, NY, USA) following the manufacturer’s instructions. The siRNA sequences used are shown in Table S1.

### Quantitative real-time PCR (qRT-PCR)

RNA isolation from kidney tissue or cells using TRIzol reagent (Invitrogen, Carlsbad, CA, USA), followed by cDNA synthesis with the ProtoScript II First Strand cDNA Synthesis Kit (E6560S, New England Biolabs, Ipswich, MA, USA). The qRT-PCR was executed on an Applied Biosystems 7500 system (Applied Biosystems, Foster City, CA, USA). Gene expression was detected using PowerUP SYBR Green Mastermix (4,367,659, ThermoFisher, Germany). The list of primers used for qRT-PCR is presented in Supplementary Table S2.

### Western blotting

The procedures were described as previously [20]. Briefly, kidney tissues or cells proteins were extracted using Radio Immuno Precipitation Assay (RIPA) lysis buffer (Beyotime Biotechnology, Shanghai, China). The protein concentration was measured using a Bicinchoninic Acid Assay (BCA) kit (Beyotime Biotechnology, Shanghai, China). Proteins were separated by SDS–PAGE and transferred to a nitrocellulose (NC) membrane. After blocking, membranes were incubated with primary antibodies at 4 °C overnight. The primary antibodies used are as follows: KIM-1 (1:1000, AF1817, R&D, USA), TNF-a (1:1000, 60291-1-Ig, proteintech, USA), IL-6 (1:1000, ab290735, Abcam, UK), Bax (1:1000, ab7977, Abcam, UK), Bcl-2 (1:1000, 68103-1-Ig, proteintech, USA), and cleaved caspase-3 (1:1000, 9664, Abcam, UK), PCNA (1:1000, ab92552, Abcam, UK), p-AKT(1:1000, 13,038 S, Cell Signaling Technology, USA), AKT(1:1000, 9272s, Cell Signaling Technology, USA).

### Chromatin Immunoprecipitation (ChIP)

ChIP was performed as previously mentioned [21]. The DNA-protein crosslinking ChIP samples from MSCs were immunoprecipitated with anti-RARα or IgG. The HAS2 primers were as follows: forward primer: 5′-CCACCTAAGGCGGAGTTCAA-3′; and reverse primer: 5′-CAGGGGACCCCACTGATTTC-3′.

### Enzyme-Linked Immunosorbent Assay (ELISA)

The concentration of hyaluronic acid (HA) in the MSCs culture supernatant was determined using an ELISA kit (DHYAL0, R&D systems, Minneapolis, MN, USA) following the manufacturer’s instructions.

### Statistical analyses

All data were shown as mean ± standard deviation (SD). Statistical analysis was performed using GraphPad Prism 8.0 (GraphPad Software, San Diego, CA, USA). Student’s t test was used for two group comparisons, and one-way ANOVA was used for multiple group comparisons. A two-sided *p*-value < 0.05 was considered statistically significant.

## Results

### ATRA pretreatment improved the renal repair effect of MSCs on AKI

**Fig. 1:**
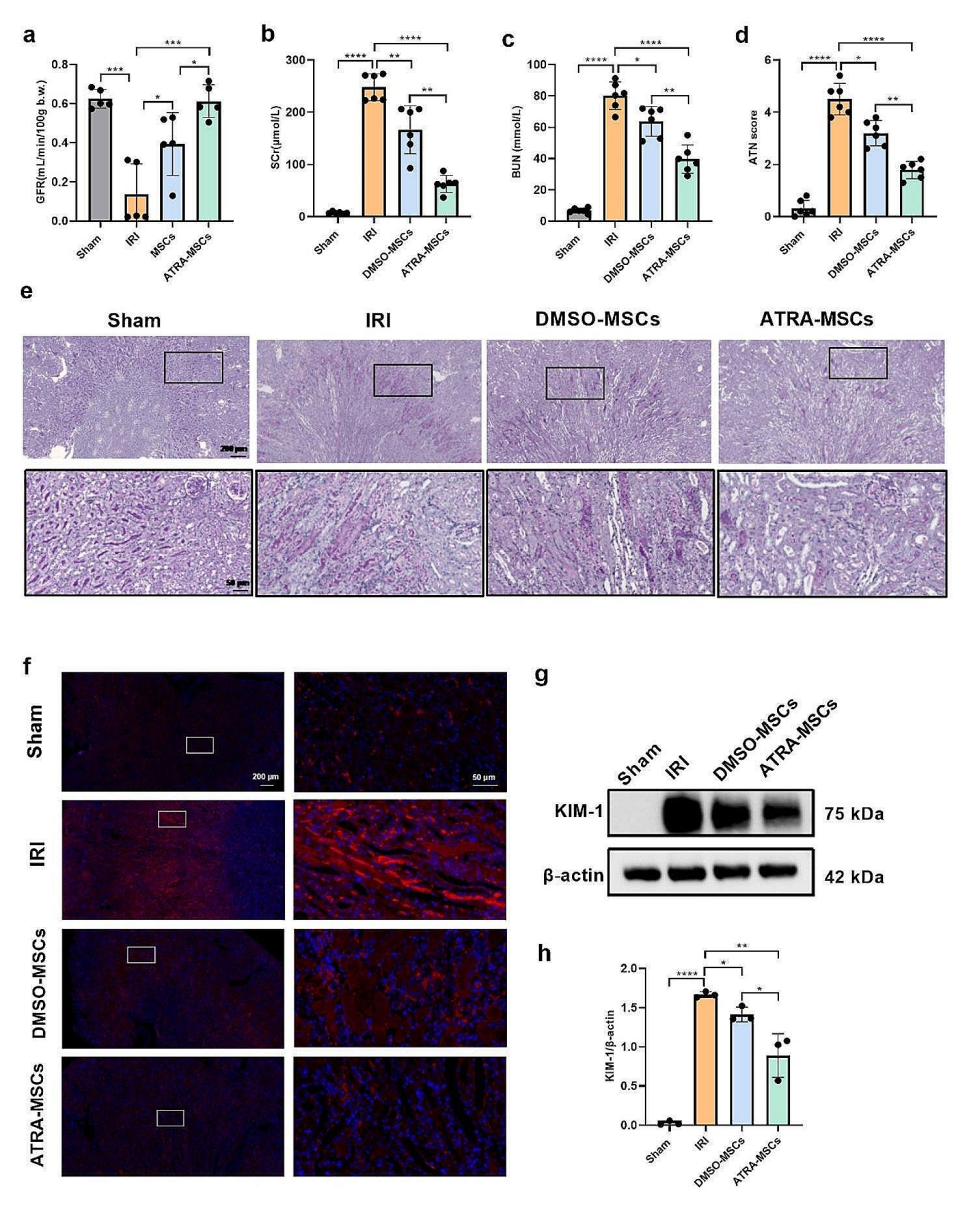
ATRA pretreatment improves the protective effect of MSCs on renal function and pathologic damage in I/R-induced AKI. a GFR assessment using transcutaneous clearance of FITC-sinistrin (*n* = 5). **b, c** SCr and BUN levels (*n* = 6). **d** Semi-quantitative analysis of histological features (*n* = 6). **e** Representative images of PAS staining on renal tissue sections in different groups of mice. Scale bars, 200 μm and 50 μm in scanned and zoom in figures, respectively. **f** Representative immunofluorescence staining of KIM-1. Scale bars, 200 μm and 50 μm in scanned and zoom in figures, respectively. **g, h** Representative western blot images and quantitative analysis of KIM-1 expression in kidney tissues (*n* = 3). Data are presented as the means ± SD. ****P < 0.0001, ***P < 0.001, **P < 0.01, *P < 0.05

We established a renal unilateral IRI model to evaluate the effectiveness of ATRA-pretreated MSCs on AKI. Mice were transplanted via tail vein with 1 × 10^6^ MSCs per mouse after reperfusion. Clearance of FITC-sinistrin was monitored transcutaneously to measure GFR, as previously described [22]. Representative tracing of FITC-sinistrin clearance in mice was shown in Supplementary Figure S1. Three days after IRI, mice exhibited a marked reduction in GFR and even a complete loss of renal function. DMSO-MSCs administration increased GFR compared with PBS-treated mice. Moreover, the GFR was further elevated with the administration of ATRA-MSCs (Fig. 1a). The mice were then sacrificed, and samples of kidney tissue and serum were collected. SCr and BUN levels are important biochemical markers to evaluate renal function. After MSCs were injected instead of PBS, the levels of SCr and BUN decreased in IRI mice. Among the therapy groups, mice injected with ATRA-MSCs exhibited significantly lower levels of SCr and BUN than those injected with DMSO-MSCs (Fig. 1b, c). PAS staining was used to assess histopathological changes. Renal tubular injury was quantified using the ATN score. In the renal IRI model, characteristic renal injuries included tubular necrosis, tubular dilation, cast formation, and brush border loss. However, ATN scores were statistically significantly reduced in the DMSO-MSCs and ATRA-MSCs groups compared to the IRI group, with the latter having the greatest decrease (Fig. 1d, e). Western blot and immunofluorescent staining results also indicated that kidney injury molecule-1(KIM-1), a biomarker of kidney tubular injury, expression in the ATRA-MSCs group was much lower than that in the DMSO-MSCs group (Fig. 1f-h). Taken together, these findings suggest that ATRA-pretreated MSCs better ameliorate AKI.

### ATRA increases HA production by upregulating HAS2 expression in MSCs

**Fig. 2:**
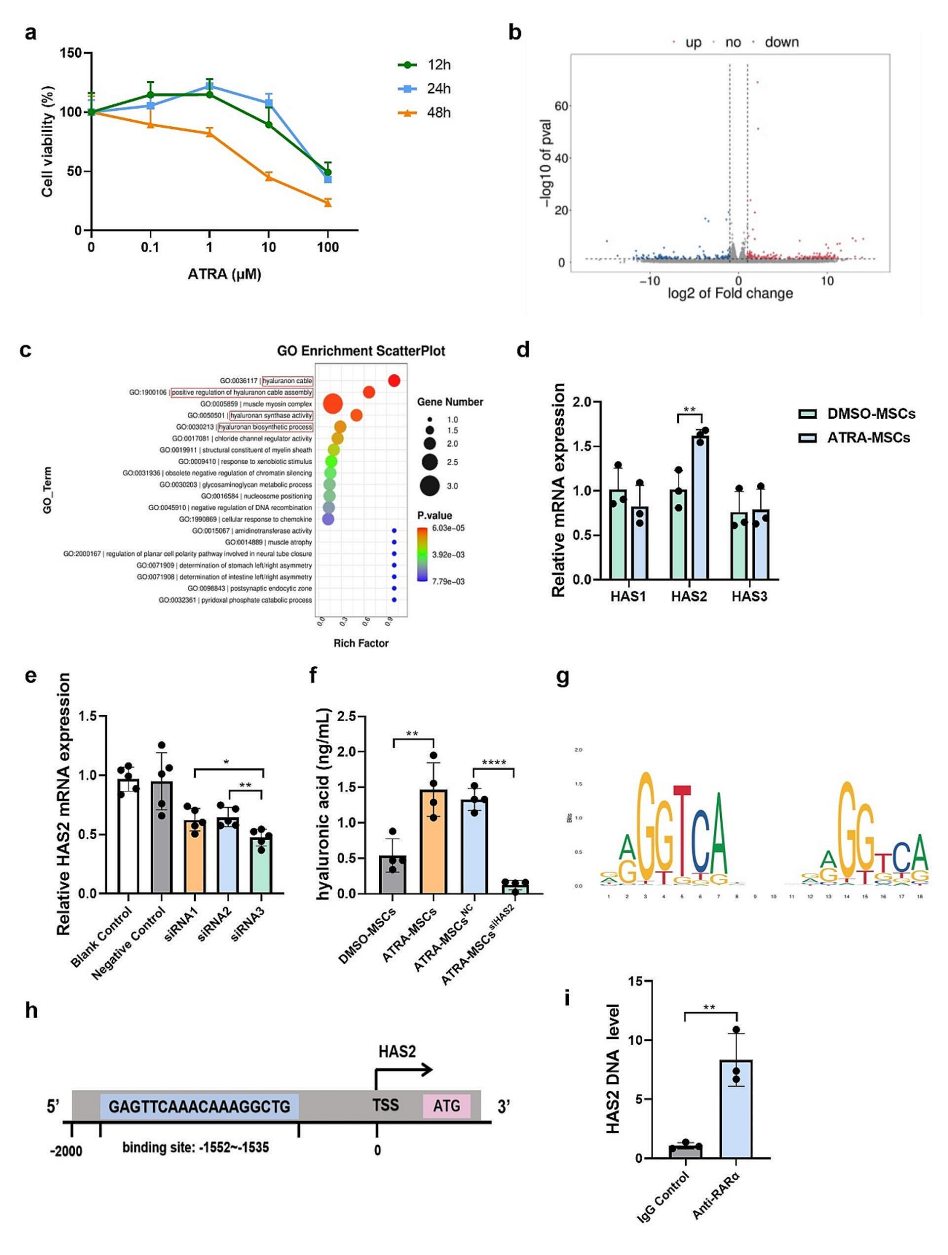
ATRA regulates the expression of HA by regulating the binding of the transcription factors RARα/RXRγ to HAS2 gene. a Viability of MSCs after ATRA pretreatment (*n* = 6). **b** Volcano plot of showing the differentially expressed genes in ATRA-MSCs versus DMSO-MSCs (*n* = 3). Red dots indicate significant up-regulation, green dots indicate significant down-regulation, and gray dots indicate insignificant differences. **c** Scatter plot of GO enrichment analysis (*n* = 3). The number of enriched differential genes can be observed by the dot size, and the significance of the enrichment is indicated by the dot color intensity. **d** The mRNA expression of HAS1, HAS2 and HAS3 (*n* = 3). **e** Transfection efficiency of different siRNA (*n* = 5). **f** The concentration of HA in the MSCs culture supernatant (*n* = 4). **g** RARα/RXRγ binding motif logo generated by the WebLogo tool. **h** Schematic representation of possible binding sites for the transcription factor RARα/RXRγ in the HAS2 promoter region. **i** ChIP-qPCR analysis of HAS2 target promotor regions (*n* = 3). Data are presented as the means ± SD. ****P < 0.0001, **P < 0.01

CCK-8 assay was used to detect the effect of ATRA on MSCs viability. Our results show that ATRA at 0.1-1 µM concentration significantly promotes the viability of MSCs at 24 h. It is notable that after 24 h of treatment, 1 µM ATRA significantly increased cell viability, while 100 µM ATRA inhibited cell viability (Fig. 2a). Accordingly, a concentration of 1 µM ATRA and a duration of 24 h were selected as the optimal intervention parameters.

RNA sequencing (RNA-seq) was used to clarify the molecular mechanisms behind the effects of ATRA pretreatment on MSCs. The volcano plot shows the differentially expressed gene (DEGs) analysis (Fig. 2b). We identified 214 up-regulated genes and 169 down-regulated genes (Supplementary Figure S2a). The Gene Ontology (GO) and Kyoto Encyclopedia of Genes and Genomes (KEGG) enrichment analysis were then used to analyze the DEGs (Supplementary Figure S2b-d). GO term enrichment analysis revealed that DEGs were mainly enriched in “ hyaluronan cable”, “positive regulation of hyaluronan cable assembly”, and “hyaluronan synthase activity” (Fig. 2c). From these results, it is clear that ATRA mainly regulates biological processes related to HA anabolism in MSCs. HA production is regulated by hyaluronan synthase genes HAS1, HAS2, and HAS3 [23]. The expression of HAS1, HAS2, and HAS3 was detected using RT-qPCR after ATRA pretreatment of MSCs. Our results showed that HAS2 expression significant up-regulated in ATRA-MSCs compared with DMSO-MSCs (Fig. 2d). After evaluating the transfection efficiency of three different siRNA, siRNA3 was selected for subsequent experiments (Fig. 2e). Similarly, ELISA showed that the supernatants of MSCs treated with ATRA produced more HA than MSCs treated with DMSO (Fig. 2f). Our data suggest that ATRA upregulates HAS2 expression, resulting in HA production by MSCs.

Many factors influence the regulation of gene expression, and one important mechanism is the binding of transcription factors to specific binding sites in gene promoters. Previous studies have identified the HAS2 as an ATRA responding gene with intricate regulatory functions [24]. Therefore, we hypothesized that some retinoic acid receptors could bind to the HAS2 promoter region and regulate HAS2 transcription. We predicted the probable transcription factors that would bind to the HAS2 promoter region by the JASPAR database, and analyzed their corresponding scores. The findings indicated that the HAS2 promoter region included several retinoic acid receptor binding sites, with RARα/RXRγ scoring the highest (Fig. 2g, h). We performed CHIP analysis to verify whether RARα/RXRγ binds to the HAS2 promoter region to promote transcription. In RXR/RAR heterodimers, RXR cannot respond to its ligand unless RAR is bound to the ligand [25]. ChIP-qPCR primers were designed to detect anti-RARα immunoprecipitated chromatin fragments based on the binding site. Notably, the HAS2 promoter region exhibited significantly higher enrichment in the RARα antibody group compared to the IgG group (Fig. 2i). Therefore, our findings suggest that the RARα/RXRγ transcription factor can bind to the HAS2 promoter, indicating that RA promotes HAS2 transcription.

### ATRA enhances the anti-inflammatory, anti-apoptosis and pro-proliferation effects of MSCs in vitro

**Fig. 3:**
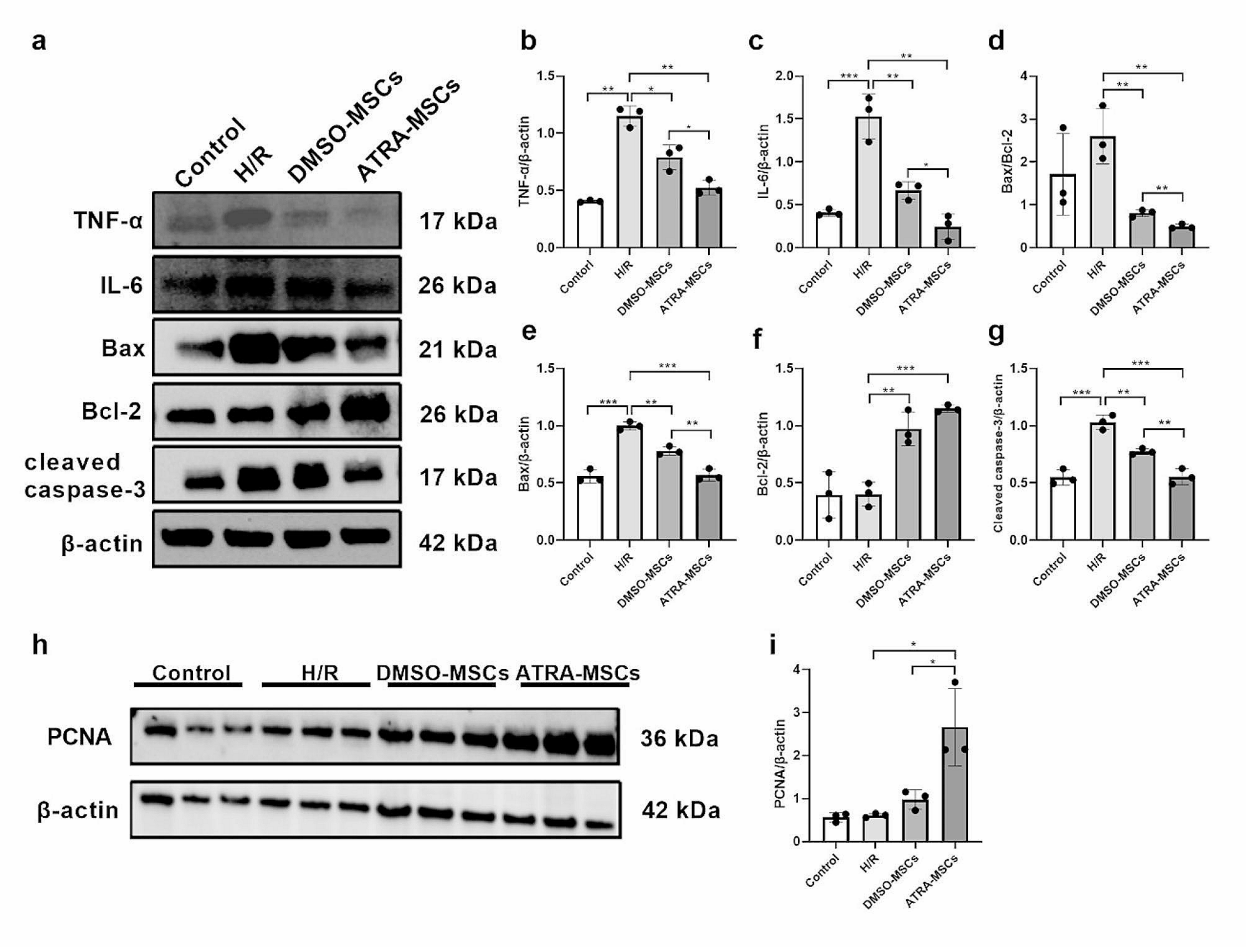
ATRA enhances the anti-inflammatory, anti-apoptosis and pro-proliferation effects of MSCs on H/R-induced HK-2 cells in vitro. a-g Representative western blot images and quantitative analysis of TNF-a, IL-6, Bax, Bcl-2, and cleaved caspase-3 in HK-2 cells (*n* = 3). **h, I** Representative western blot images and quantitative analysis of PCNA in HK-2 cells (*n* = 3). Data are presented as the means ± SD. ***P < 0.001, **P < 0.01, *P < 0.05

Inflammation and apoptosis play an essential role in the development of AKI [26]. Recent studies have shown that stem cells possess antioxidant, immunomodulatory and anti-apoptotic properties, making them a promising therapeutic tool for AKI [27]. To explore the effect of ATRA-MSCs on AKI, we constructed an in vitro model of H/R-induced HK-2 cells to simulate renal IRI. HK-2 cells were co-cultured with DMSO-MSCs or ATRA-MSCs for 24 h after hypoxia. Western blot was conducted to measure inflammation-related proteins (TNF-a and IL-6) and apoptosis-related proteins (Bax, Bcl-2, and cleaved caspase-3) levels in HK-2 cells. The results show that the expression of TNF-a and IL-6 was significantly reduced in H/R-induced HK-2 cells after co-culture with DMSO-MSCs or ATRA-MSCs. Moreover, compared with the DMSO-MSCs group, the ATRA-MSCs group exhibited substantially lower protein expression levels of TNF-α and IL-6. Additionally, we examined the role of MSCs in cellular apoptosis. The results demonstrate that the expression of Bax and cleaved caspase-3 proteins was significantly decreased, while the expression of Bcl-2 protein was significantly increased in HK-2 cells co-cultured with DMSO-MSCs or ATRA-MSCs. The improvement of apoptosis in H/R-induced HK-2 cells was more pronounced after ATRA pretreatment with MSCs (Fig. 3a-g). Renal tubular cell proliferation and repair are recognized as key processes in renal recovery after AKI [28]. We observed that PCNA protein expression of HK-2 cells was increased after co-culture with DMSO-MSCs or ATRA-MSCs, with the latter increasing more significantly (Fig. 3h, i). From the results above, we found that ATRA pretreatment significantly enhanced the anti-inflammatory, anti-apoptotic and proliferative repair effects of MSCs on AKI in vitro.

### HA/cluster determinant 44 (CD44) axis activated the PI3K/AKT signaling pathway to promote renal repair

**Fig. 4:**
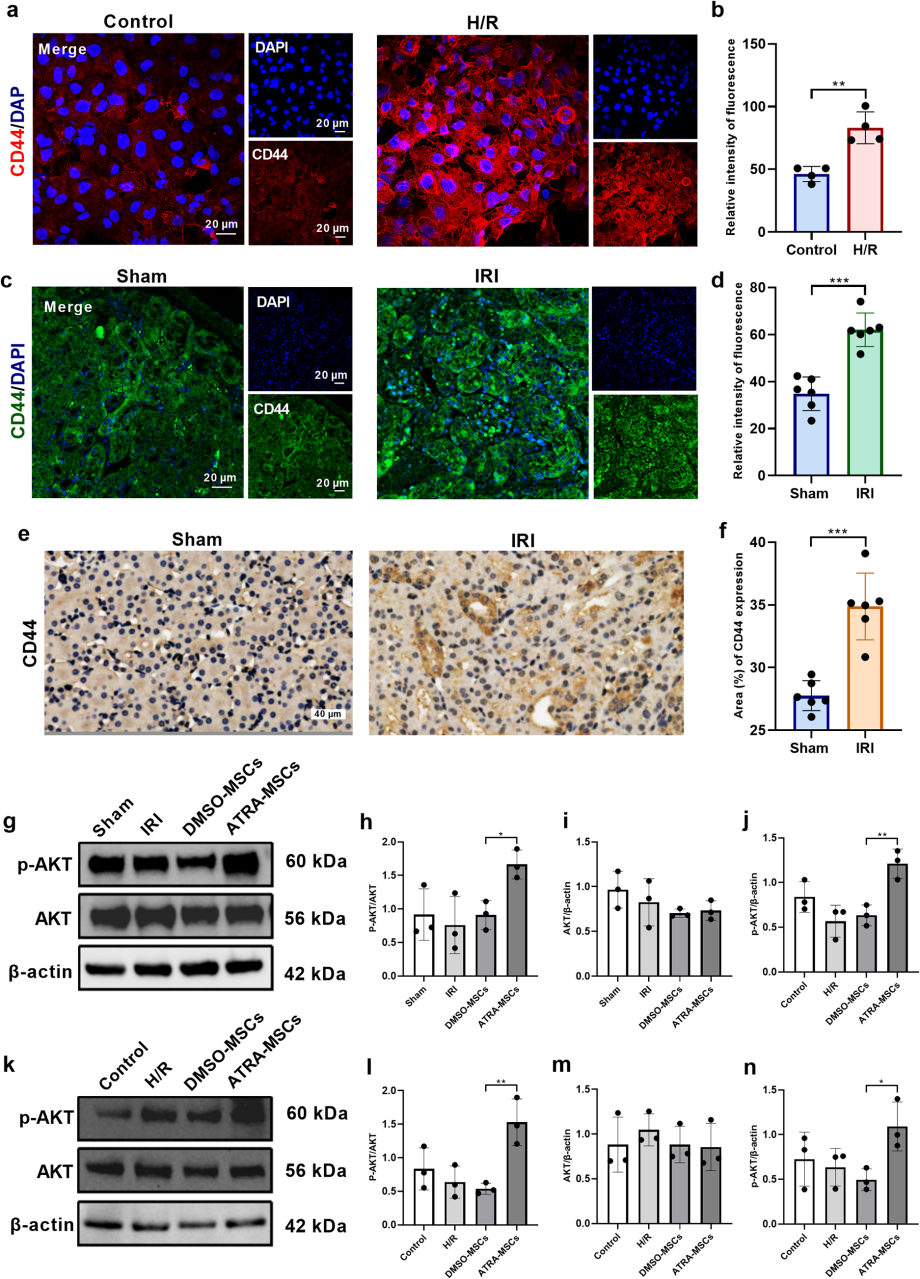
HA activates the downstream PI3K/AKT signaling pathway by binding to CD44. **a, b** Representative images and quantification of immunofluorescence staining of HK-2 cells with CD44 (red) and DAPI (blue) (*n* = 3). Scale bars, 20 μm. **c, d** Representative immunofluorescence staining of CD44 in renal tissue (*n* = 6). Scale bars, 20 μm. **e, f** Representative immunohistochemical staining of CD44 in renal tissue (*n* = 6). Scale bars, 40 μm. **g-j** Representative western blot images and quantitative analysis of p-AKT and total AKT levels in kidney tissues (*n* = 3). **k-n** Representative western blot images and quantitative analysis of p-AKT and total AKT levels in HK-2 cells (*n* = 3). Data are presented as the means ± SD. **P < 0.01, *P < 0.05

CD44 is the main receptor for HA [29]. Immunofluorescence analysis revealed higher expression of CD44 in H/R-induced HK-2 cells than in normal HK-2 cells (Fig. 4a, b). Furthermore, the expression of CD44 was also significantly increased in kidney tissues of IRI mice compared with those of sham-operated mice (Fig. 4c-f). Previous studies have demonstrated that HA can promote cell growth and migration by activating the PI3K/AKT signaling pathway [30]. To verify whether ATRA-pretreated MSCs exert nephroprotective effects by activating the PI3K/AKT pathway, western blot was conducted to detect phospho-AKT (p-AKT) and total AKT protein levels. The results showed that the expression of total AKT and p-AKT in renal tissues of AKI mice did not change significantly after MSCs treatment, while the expression of p-AKT increased after MSCs pretreated with ATRA (Fig. 4g-j). Interestingly, in co-culture experiments, we observed that MSCs pretreated with ATRA increased H/R-induced AKT phosphorylation in HK-2 cells. In contrast, p-AKT levels in HK-2 cells co-cultured with DMSO-pretreated MSCs did not significantly change (Fig. 4k-n). These findings suggest that ATRA can promote HA production, thereby activating the PI3K/AKT signaling pathway to exert renoprotective effects.

### ATRA pretreatment of MSCs attenuates inflammation and apoptosis and promotes proliferative renal repair in AKI

**Fig. 5:**
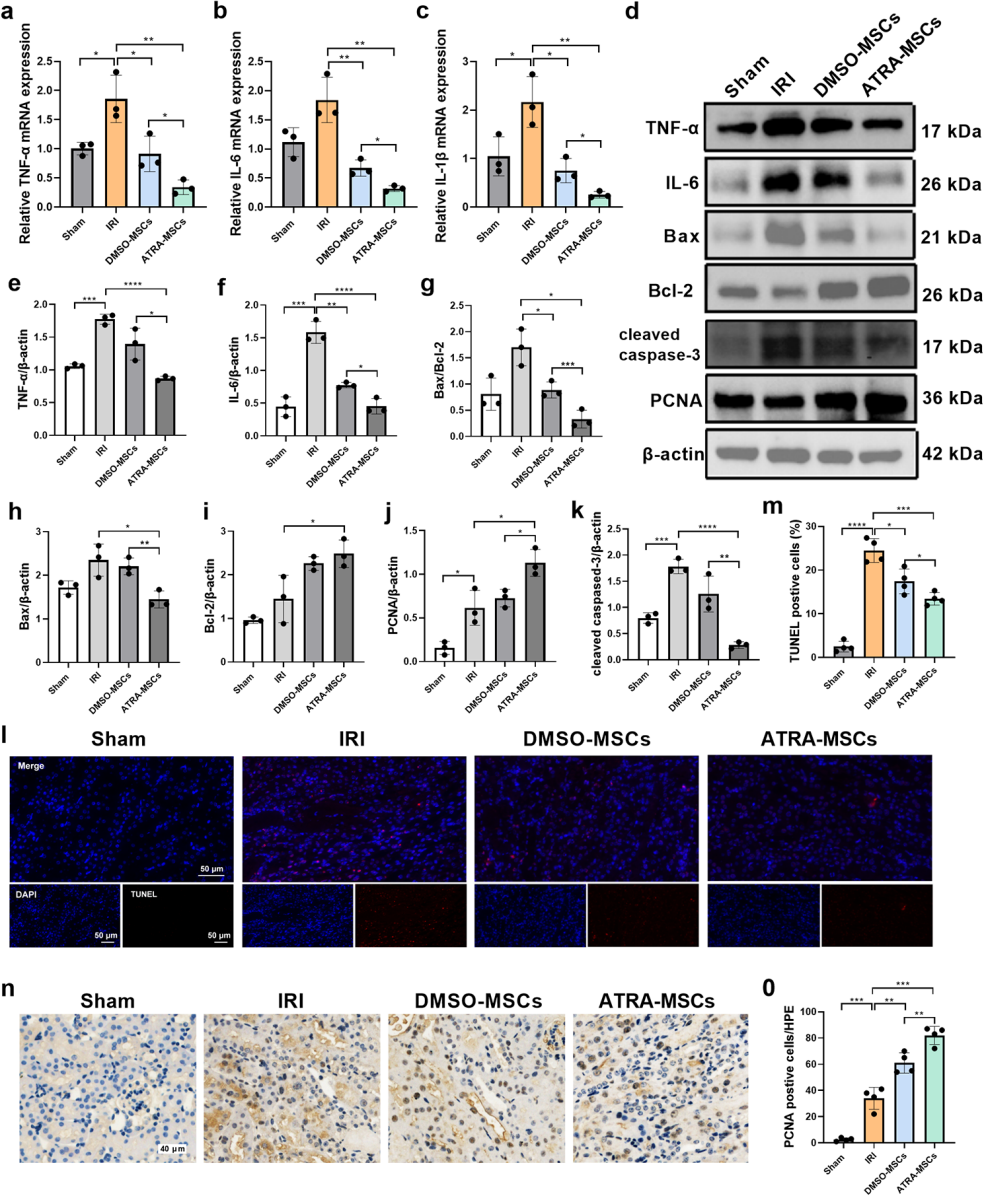
ATRA enhances the anti-inflammatory, anti-apoptosis and pro-proliferation effects of MSCs on I/R-induced AKI in vivo. **a** TNF-a mRNA expression level (*n* = 3). **b** IL-6 mRNA expression level (*n* = 3). **c** IL-1β mRNA expression level (*n* = 3). **d-j** Representative western blot images and quantitative analysis of TNF-a, IL-6, Bax, Bcl-2, cleaved caspase-3 and PCNA in kidney tissues (*n* = 3). **k** Apoptotic cells were labeled with the TUNEL assay kit (red) with nuclei counterstained with DAPI (blue) in kidney sections. Scale bar, 50 μm. **l** Quantitative analysis of the percentage of apoptotic cells (*n* = 3). **m** Representative images of PCNA in kidney tissues. Scale bar, 40 μm. **n** Quantitative analysis of PCNA-positive cells (*n* = 3). Data are presented as the means ± SD. ****P < 0.0001, ***P < 0.001, **P < 0.01, *P < 0.05

We demonstrated that ATRA-pretreated MSCs improve renal function parameters in AKI mice. Based on our findings in vitro, we confirmed the effect of ATRA-pretreated MSCs on inflammation, apoptosis, and proliferation of hypoxic HK-2 cells. In I/R-induced mice, decreased mRNA levels of TNF-a, IL-6, and IL-1β were shown in the ATRA-MSCs group and the DMSO-MSCs group. However, TNF-a, IL-6, and IL-1β mRNA expression was dramatically lower the ATRA-MSCs group in contrast to the DMSO-MSCs group (Fig. 5a-c). Similarly, TNF-a and IL-6 expression in the ATRA-MSCs group was significantly lower than in the DMSO-MSCs group, according to western blot analysis (Fig. 5d-f). Additionally, transplantation of DMSO-MSCs or ATRA-MSCs decreased the expression levels of Bax and cleaved caspase-3, while increasing the expression level of Bcl-2. Notably, the ATRA-MSCs group exhibited a superior therapeutic effect on reducing apoptosis than the DMSO-MSCs group (Fig. 5d, g-i). Consistently, TUNEL labeling of the ATRA-MSCs group revealed fewer apoptotic cells than in the DMSO-MSCs and PBS groups. (Fig. 5k, l). The results of immunohistochemical staining and western blot demonstrated that the expression of proliferation-related PCNA was significantly increased in renal tissues after DMSO-MSCs or ATRA-MSCs transplantation, with higher expression observed in the ATRA-MSCs group (Fig. 5d, j, m, n). Taken together, ATRA-MSCs attenuated inflammation and apoptosis, and promoted the proliferation of renal tubular epithelial cells in IRI mice.

### Inhibition of the HA/CD44 axis reversed the renal repair effect of ATRA-pretreated MSCs

**Fig. 6:**
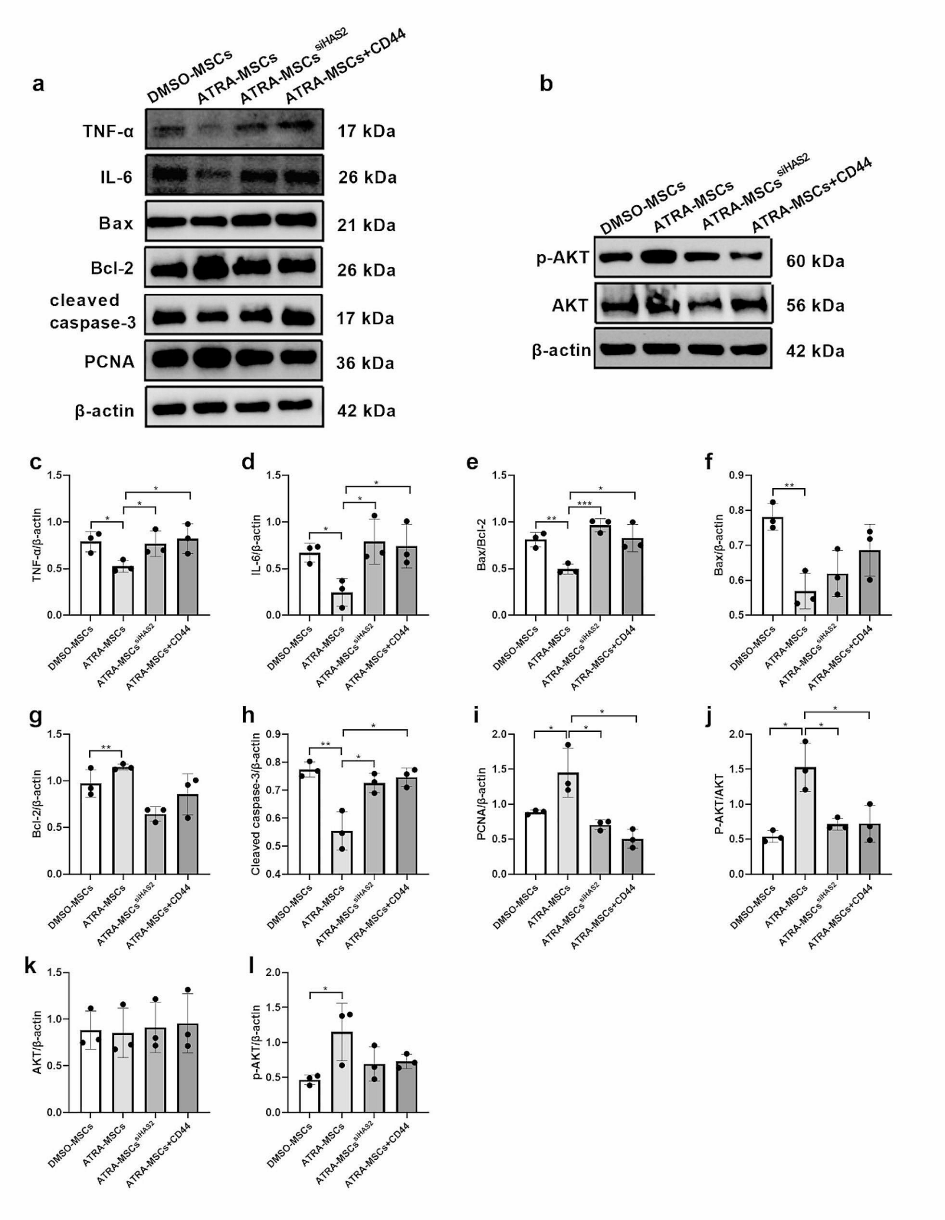
Inhibition of the HA/CD44 axis reverses the anti-inflammatory, anti-apoptotic and pro-proliferative effects of ATRA-MSCs. **a, c-i** Western blot images and quantitative densitometry analysis of TNF-a, IL-6, Bax, Bcl-2 and cleaved caspase-3 in HK-2 cells (*n* = 3). **b, j-l** Western blot images and quantitative densitometry analysis of p-AKT and total AKT in HK-2 cells (*n* = 3). Data are presented as the means ± SD. ****P < 0.0001, ***P < 0.001, **P < 0.01, *P < 0.05

We further investigated whether ATRA-pretreated MSCs exert renal repair effects on AKI through the HA/CD44 axis. To block HA binding to CD44 on HK-2 cells, a soluble CD44 blocking antibody was added to the HK-2 cells culture medium for 2 h before co-culture with MSCs. Simultaneously, HAS2 expression was knocked down in MSCs using small interfering RNA (siRNA). ATRA-pretreated MSCs significantly reduced HK-2 cells inflammation and apoptosis, and promoted cells proliferation. However, silencing HAS2 or blocking CD44 increased the protein expression of TNF-a, IL-6, Bax, and cleaved caspase-3 and decreased the protein expression of Bcl-2 and PCNA (Fig. 6a, c-i). In other words, the anti-inflammatory, anti-apoptosis and pro-proliferation effects of ATRA-MSCs were weakened after silencing HAS2 or blocking CD44. Furthermore, we found that ATRA-pretreated MSCs activated the PI3K/AKT pathway in H/R-induced HK-2 cells, whereas HAS2 silencing or CD44 antibody blockade effectively reduced AKT phosphorylation (Fig. 6b, j-l). These results indicate that inhibition of the HA/CD44 axis reverses the renal repair effect of MSCs pretreated with ATRA on AKI.

**Fig. 7:**
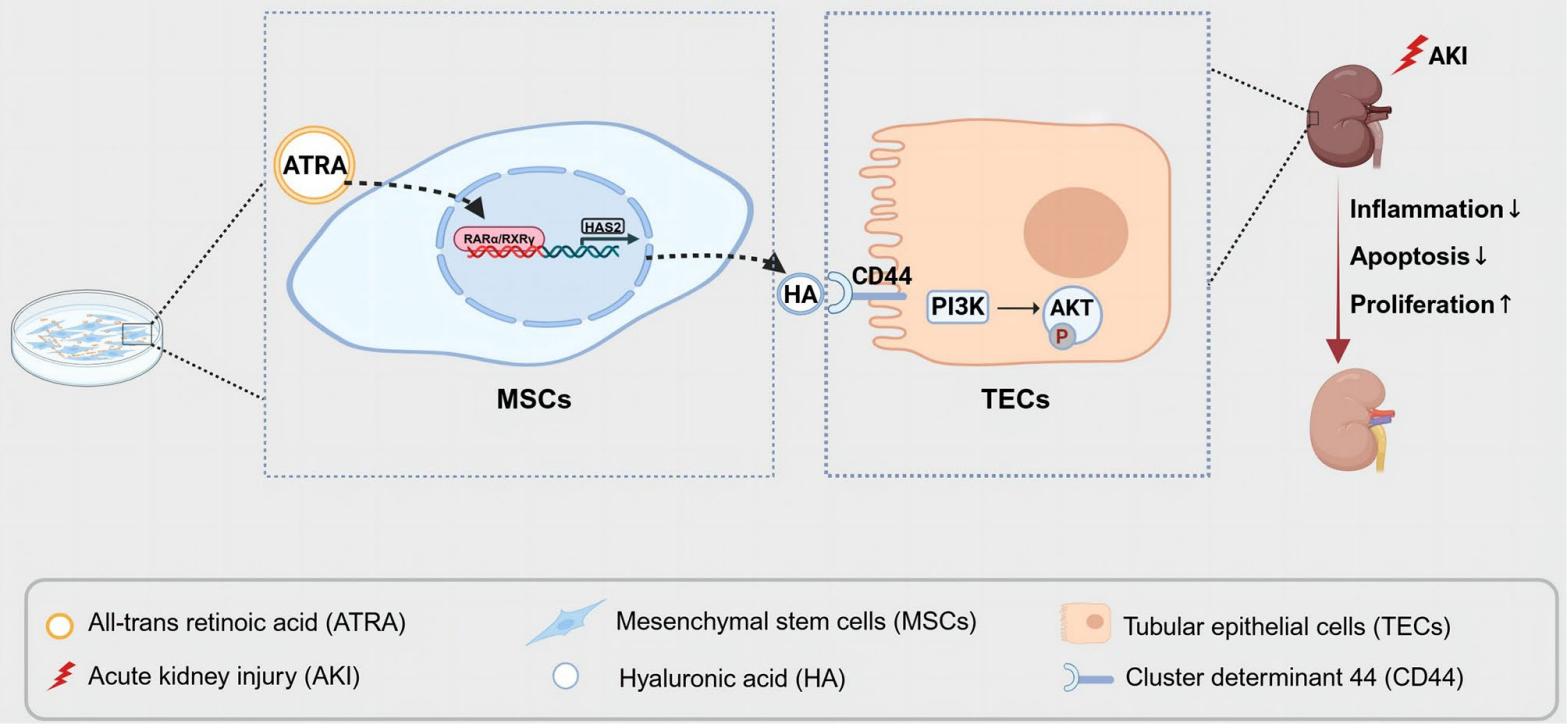
Schematic illustration of ATRA promoted HA production by MSCs and activated the PI3K/AKT pathway by binding to CD44 in renal tubular epithelial cells, thereby improving renal repair after AKI

## Discussion

MSCs are widely used in regenerative medicine, exerting substantial benefits in the treatment of AKI [31]. More and more pretreatment strategies have emerged to improve the survival rate, homing rate, and efficacy of MSCs [32, 33]. In this research, we observed that ATRA pretreatment of MSCs effectively ameliorated renal function and reduced renal pathological damage in AKI. Meanwhile, studies conducted in vitro and in vivo demonstrated that MSCs pretreatment with ATRA had positive effects on AKI in terms of anti-inflammatory responses, anti-apoptotic, and proliferative repair. In addition, we identified retinoic acid receptors as potential transcription factors of HAS2 that activate HAS2 transcription and promote HA production. HA interacts with CD44 to activate the PI3K/AKT signaling pathway, ultimately reducing kidney injury. Additionally, silencing HAS2 or blocking CD44 could reverse the nephroprotective effect of ATRA-pretreated MSCs. Our study demonstrates that the nephroprotective effect of ATRA-pretreated MSCs is mediated by activation of the PI3K/AKT pathway through the HA/CD44 axis (Fig. 7).

ATRA can enhance the effect of MSCs on wound healing by inducing various chemokine receptors and proangiogenic factors. Cyclooxygenase-2 (COX-2) expression and Prostaglandin E2 (PGE_2_) levels were significantly elevated in ATRA-pretreated MSCs [14]. Han et al. demonstrated that ATRA-MSCs therapy exhibited more benefits than MSCs in treating spinal cord injury by inhibiting excessive inflammation and activating autophagy [15]. Interestingly, we also found that ATRA markedly improved MSCs effectiveness in AKI. Mice treated with ATRA-MSCs had dramatically lower levels of SCr, BUN, and ATN scores and much higher levels of GFR than mice treated with DMSO-MSCs. Compared with DMSO-MSCs treatment, ATRA-MSCs treated mice had significantly lower KIM-1 expression, reduced inflammation and apoptosis, and enhanced renal regenerative repair.

ATRA-pretreated MSCs have beneficial therapeutic effects in terms of ameliorating inflammation, apoptosis and oxidative stress in AKI, but the mechanism of this renal repair has not been fully elucidated [34, 35]. Hence, we further investigated the potential mechanisms of ATRA impact on MSCs using RNA-seq. We found that differential genes were mainly enriched in HA anabolism. HA synthesis and degradation are regulated by the hyaluronan synthases (HAS1, HAS2, and HAS3) and the hyaluronidases (HYAL1 and HYAL2) [23]. Our results found that HAS2 expression was upregulated, and HA production increased after ATRA pretreatment of MSCs. Retinoic acid (RA) is an active metabolite of vitamin A, and its signaling is mediated mainly by two families of nuclear receptors: retinoic acid receptors (RARα, RARβ, and RARγ) and retinoid X receptors (RXRα, RXRβ, and RXRγ). These two receptors often form RAR/RXR heterodimers that bind to retinoic acid response elements (RAREs) in the promoter regions of target genes. When the ATRA binds to the receptor, it results in a conformational change that leads to the release of the co-repressor complex and the recruitment of the co-activator complex, eventually activating gene transcription [36]. HAS2 was found to be an ATRA responsive gene with functional RAREs cluster upstream of the transcription start site [24, 37]. Therefore, we performed prediction analysis using the JASPAR database and discovered multiple RA receptor binding sites in the promoter region of the HAS2. Subsequently, we validated that RARα/RXRγ could bind to the HAS2 promoter using the ChIP-qPCR. These results suggest that the HAS2 gene is actively regulated by RARα/RXRγ.

HA is a non-sulfated glycosaminoglycan component of the extracellular matrix, composed of repeating disaccharide subunits of N-acetylglucosamine and β-glucuronic acid. Due to their negatively charged polymer chains, HA molecules assume a fully unfolded conformation in water, occupying significant space and enabling HA to absorb large amounts of water, resembling a sponge. HA is essential for maintaining tissue hydration, elasticity, and lubrication. Moreover, it is pivotal in promoting wound healing, tissue regeneration, and ameliorating inflammation [23, 38]. IL-10 was found to regulate the metabolism of HA and stimulate fibroblasts to produce HA-rich pericellular matrices, which attenuate fibrotic and inflammatory dysregulation in wound healing models [39]. In a unilateral ureteral obstruction model, IL-10 overexpression promoted HA synthesis and reduced renal fibrosis, which could be inhibited by HA synthesis inhibitor, 4-methylumbelliferone (4-MU) [40]. HA interacts with many proteins, of which CD44 is the main signal-transducing receptor [29]. CD44 is a cell surface glycoprotein that is expressed in many cell types, such as lymphocytes, fibroblasts, smooth muscle cells, and tumor cells. The main functions of CD44 include intercellular adhesion, cell migration, and cell proliferation [41, 42]. In line with earlier research, we observed a significant increase of CD44 expression in renal tissues of IRI mice [43]. CD44-deficient mice showed more severe tubular injury in obstructive nephropathy, which was attributed to lower tubular epithelial cell proliferation and greater apoptosis. However, these mice also promoted renal fibrogenesis partly through higher HGF and TGF-β1 signaling pathways [44]. Several studies have clearly shown that HA activates the PI3K/AKT signaling pathway, which then promotes cell survival. Interfering with HA-CD44 binding leads to suppression of the PI3K/AKT cell survival pathway while inhibiting tumor growth [30, 45]. In our study, ATRA pretreatment of MSCs activated the PI3K/AKT signaling pathway in AKI. Inhibition of HAS2 expression or blocking CD44 binding did not activate the PI3K/AKT signaling pathway when hypoxic HK-2 cells were co-cultured with ATRA-pretreated MSCs. Remarkably, the anti-inflammatory, anti-apoptosis and pro-proliferation effects of ATRA-pretreated MSCs were weakened by inhibiting HA/CD44 axis. Taken together, these results suggest that the protective effect of ATRA-pretreated MSCs in AKI is mediated by HA/CD44 axis.

As MSCs are highly sensitive to the microenvironment, the rational use of small molecule compounds can optimize the therapeutic effect of stem cells [46]. Our study observed that ATRA pretreatment of MSCs enhanced the renoprotective effect in AKI by HA production, but the other mechanisms of the protective effect remain unclear. Studies have shown that ATRA can also secrete cytokines, activate the retinoid signaling pathway, and regulate immunity, contributing to the efficacy of cells [47–50]. In the future, the optimization strategy and new therapeutic targets of MSCs therapy still need to be further studied.

In conclusion, our study demonstrates that ATRA promoted HA production by MSCs and activated the PI3K/AKT pathway by binding to CD44 in renal tubular epithelial cells, thereby improving renal repair after AKI.

### Electronic supplementary material

Below is the link to the electronic supplementary material.

Supplementary Material 1

Supplementary Material 2

## Data Availability

Not applicable.

## Abbreviations

AKI: Acute kidney injury
IRI: Ischemia-reperfusion injury
H/R: Hypoxia/reoxygenation
ATRA: All-trans retinoic acid
MSCs: Mesenchymal stem cells
GFR: Glomerular filtration rate
SCr: Serum creatinine
BUN: Blood urea nitrogen
ATN: Acute tubular necrosis
HA: Hyaluronic acid, HAS: Hyaluronic acid synthase
CD44: Cluster determinant 44
