# Supplementary material for: All-trans retinoic acid pretreatment of mesenchymal stem cells enhances the therapeutic effect on acute kidney injury
Source: Cell Commun Signal. 2024 May 27;22:291. doi: 10.1186/s12964-024-01671-1 (PMC11129434; doi:10.1186/s12964-024-01671-1)
Supplement: Supplementary file 1 — Supplementary Material 1 [file 12964_2024_1671_MOESM1_ESM.docx]

**All-trans retinoic acid pretreatment of mesenchymal stem cells enhances the therapeutic effect on acute kidney injury**

Yifan Zhang^1,2†^, Xiaochen Wang^1,2†^, Yuwei Ji^1,2†^, Haijuan Qong^3^, Xiaodong Geng^2,4^, Keying Zhang^1,2^, Zhangning Fu^1,2^, Guangyan Cai^2^, Xiangmei Chen^2^, Ping Li^2*^, Quan Hong^2*^ ^1^Department of Nephrology, First Medical Center of Chinese PLA General Hospital, National Key Laboratory of Kidney Diseases, National Clinical Research Center for Kidney Diseases, Beijing Key Laboratory of Kidney Diseases Research, Beijing 100853, China

^2^Medical School of Chinese PLA, Beijing, 100853, China.

^3^Songjiang District Central Hospital, Shanghai, China.

^4^Health Care Office of the Service Bureau of Agency for Offices Administration of the Central Military Commission, Beijing, China.

**^#^Yi-fan Zhang, Xiao-chen Wang, Yu-wei Ji contributed equally to this article and shared first authorship.**

**Correspondence:**

*Quan Hong

Email: hongquan@301hospital.com.cn

Affiliation: Department of Nephrology, First Medical Center of Chinese PLA General Hospital, National Key Laboratory of Kidney Diseases, National Clinical Research Center for Kidney Diseases, Beijing Key Laboratory of Kidney Diseases Research, Beijing 100853, China

Address: First Medical Center of Chinese PLA General Hospital, No.28 Fuxing Road, Beijing 100853, China.

*Ping Li

Email: liping.8@163.com

Affiliation: Department of Nephrology, First Medical Center of Chinese PLA General Hospital, National Key Laboratory of Kidney Diseases, National Clinical Research Center for Kidney Diseases, Beijing Key Laboratory of Kidney Diseases Research, Beijing 100853, China

Address: First Medical Center of Chinese PLA General Hospital, No.28 Fuxing Road, Beijing 100853, China.

***Quan Hong and Ping Li shared the corresponding authorship in this work.**

**Supplemental methods**

**Supplemental figures**

**
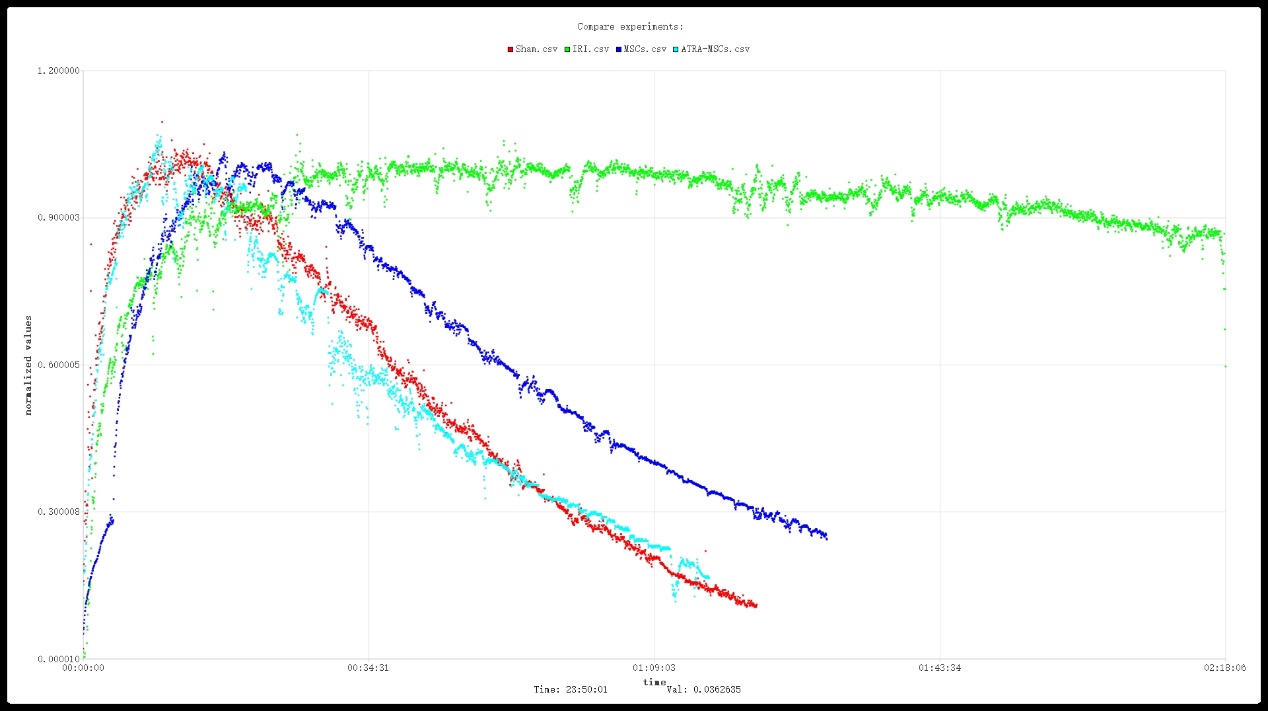
**

**Supplementary Figure S1.** Representative tracing of FITC-sinistrin clearance in mice

**
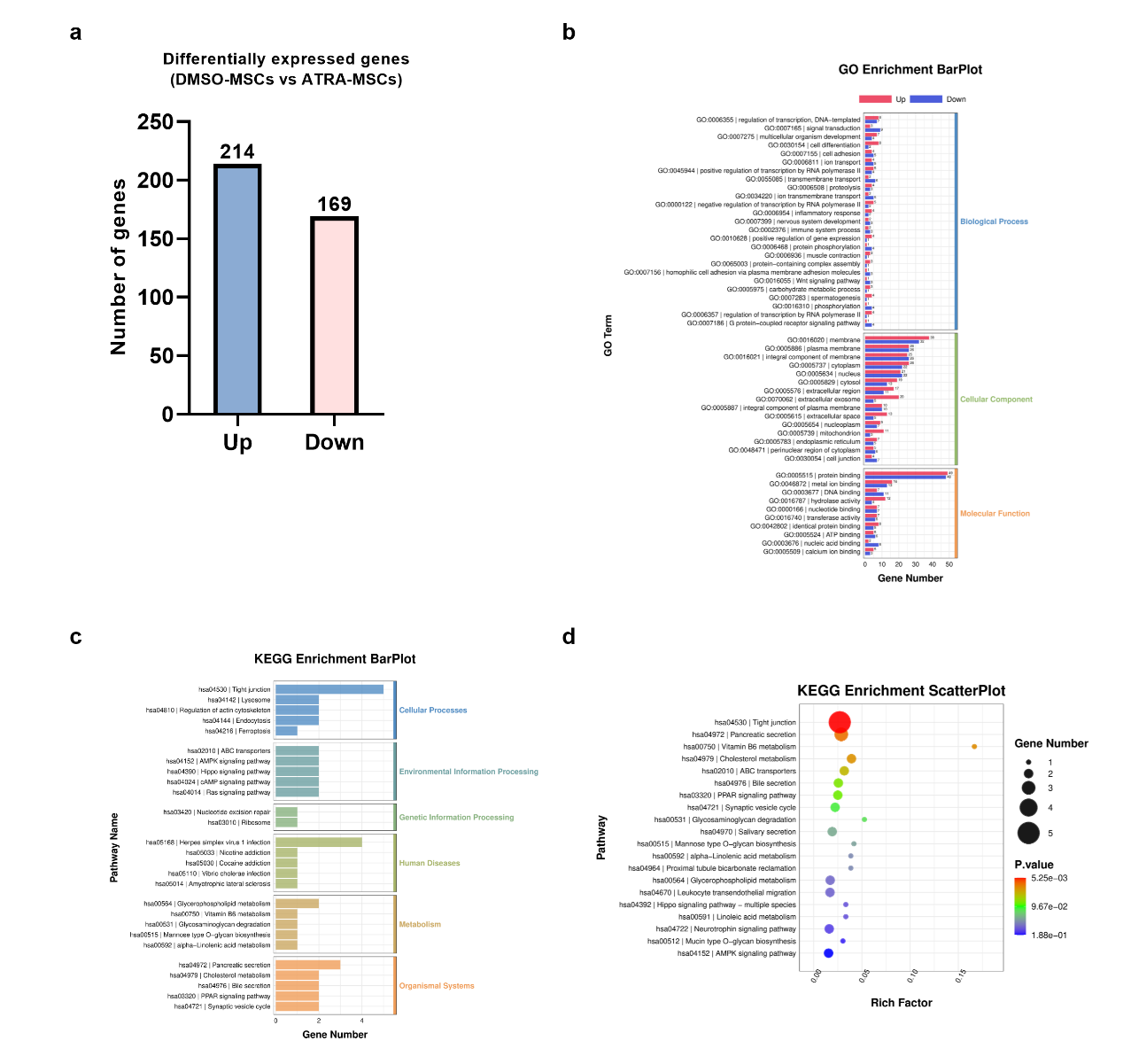
**

**Supplementary Figure S2.** Differential expression genes (DEGs) enrichment analysis. **a** The number of differentially expressed genes in the DMSO-MSCs group and the ATRA-MSCs group. **b** GO enrichment analysis was divided into three main categories: biological process, cellular component, and molecular function. **c** KEGG pathway analysis was divided into six main categories: cellular Process, environmental Information Process, genetic Information Process, metabolism, human Diseases, and organismal Systems. **d** Scatter plot of differential expression gene KEGG enrichment analysis.

**Supplementary Tables**

**Supplementary Table S1. Sequences of siRNAs used.**

| **Gene** | **Forward Primer Sequences** | **Reverse Primer Sequences** |
| --- | --- | --- |
| HAS2 | GCCACUUAUAUCUGGAAGATT | UCUUCCAGAUAUAAGUGGCTT |
| Negative control (Non-targeting) | UUCUCCGAACGUGUCACGUTT | ACGUGACACGUUCGGAGAATT |

**Supplementary Table S2. Primer sequences used for qRT-PCR analyses.**

| **Gene** | **Forward Primer Sequences** | **Reverse Primer Sequences** |
| --- | --- | --- |
| HAS1 | ATGGGTTATGCTACCAAGTACACC | GCCACTCACGGAAGTACGAC |
| HAS2 | GGCCGGTCGTCTCAAATTCAT | ACCTCTCACAATGCATCTTGTTC |
| HAS3 | ATCATGCAGAAGTGGGGAGGC | TCCAGGACTCGAAGCATCTC |
| TNF-a | CACAGTGAAGTGCTGGCAAC | GATCAAAGCTGTAGGCCCCA |
| IL-6 | CCACCGGGAACGAAAGAGAA | GAGAAGGCAACTGGACCGAA |
| IL-1β | CAGGCAGGCAGTATCACTCA | TGTCCTCATCCTGGAAGGTC |
| GAPDH | TGCACCACCAACTGCTTAGC | GGCATGGACTGTGGTCATGAG |
