# Supplementary material for: All-trans retinoic acid pretreatment of mesenchymal stem cells enhances the therapeutic effect on acute kidney injury
Source: Cell Commun Signal. 2024 May 27;22:291. doi: 10.1186/s12964-024-01671-1 (PMC11129434; doi:10.1186/s12964-024-01671-1)
Supplement: Supplementary file 2 — Supplementary Material 2 [file 12964_2024_1671_MOESM2_ESM.pdf]

Figure 1

Sham IRI DMSO-MSCs ATRA-MSCs / Sham IRI DMSO-MSCs ATRA-MSCs / Sham IRI DMSO-MSCs ATRA-MSCs

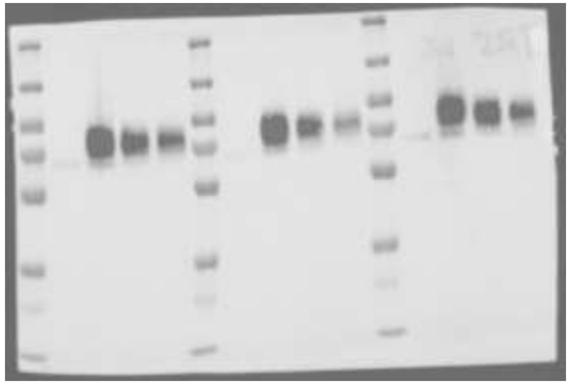

KIM-1 (75 KDa)

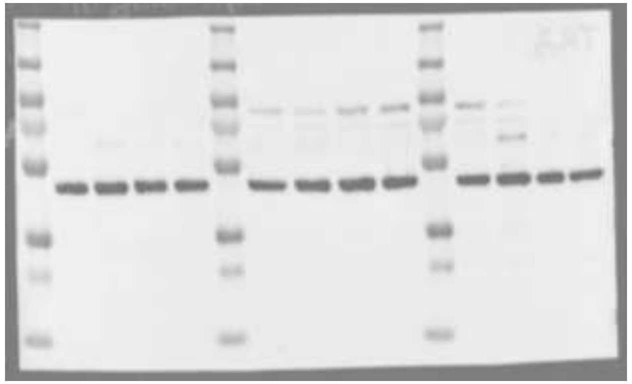

β-actin (42 KDa)

Figure 3

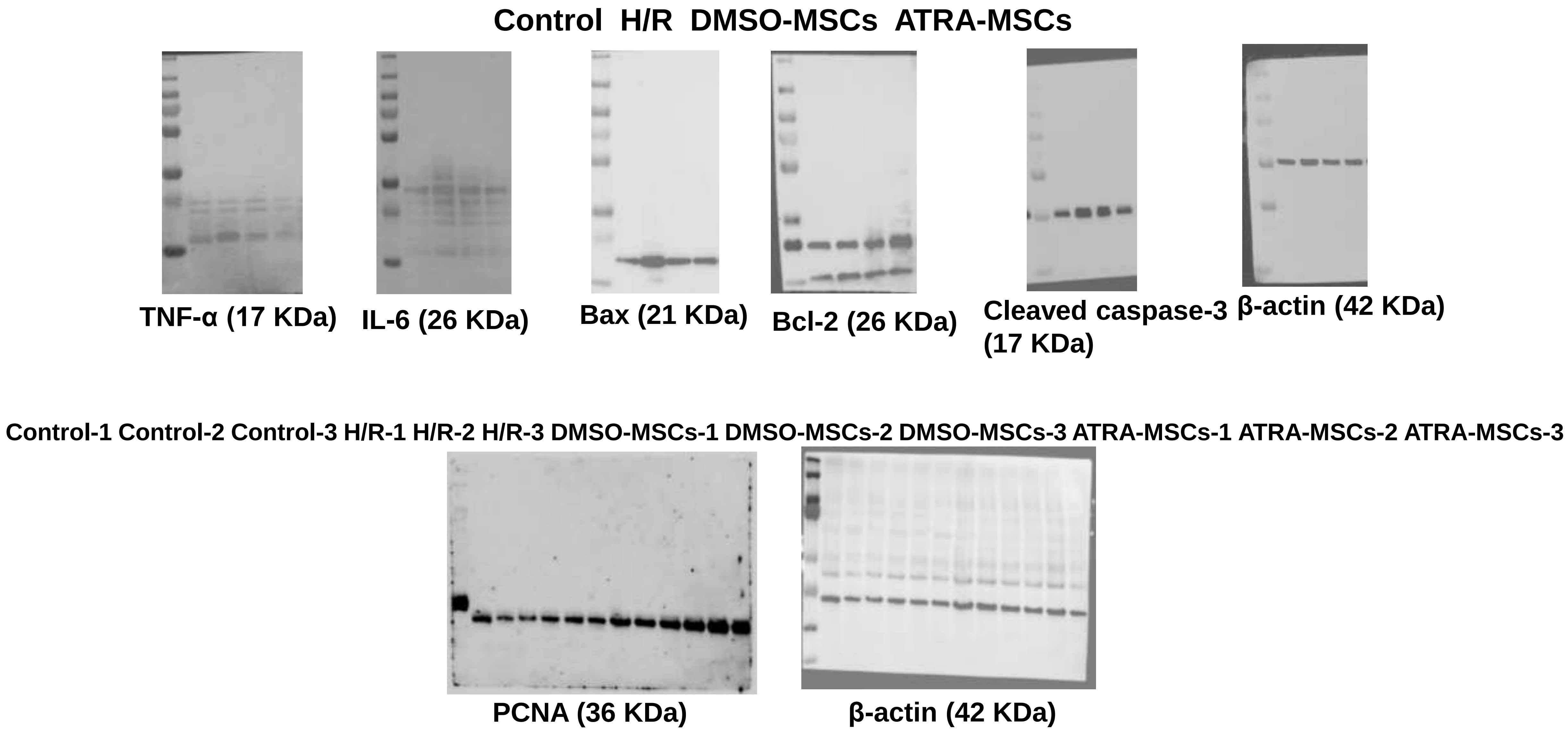
Control H/R DMSO-MSCs ATRA-MSCs
TNF-α (17 KDa)
IL-6 (26 KDa)
Bax (21 KDa)
Bcl-2 (26 KDa)
Cleaved caspase-3 (17 KDa)
β-actin (42 KDa)
Control-1 Control-2 Control-3 H/R-1 H/R-2 H/R-3 DMSO-MSCs-1 DMSO-MSCs-2 DMSO-MSCs-3 ATRA-MSCs-1 ATRA-MSCs-2 ATRA-MSCs-3
PCNA (36 KDa)
β-actin (42 KDa)

Figure 4

Sham IRI DMSO-MSCs ATRA-MSCs / Sham IRI DMSO-MSCs ATRA-MSCs / Sham IRI DMSO-MSCs ATRA-MSCs

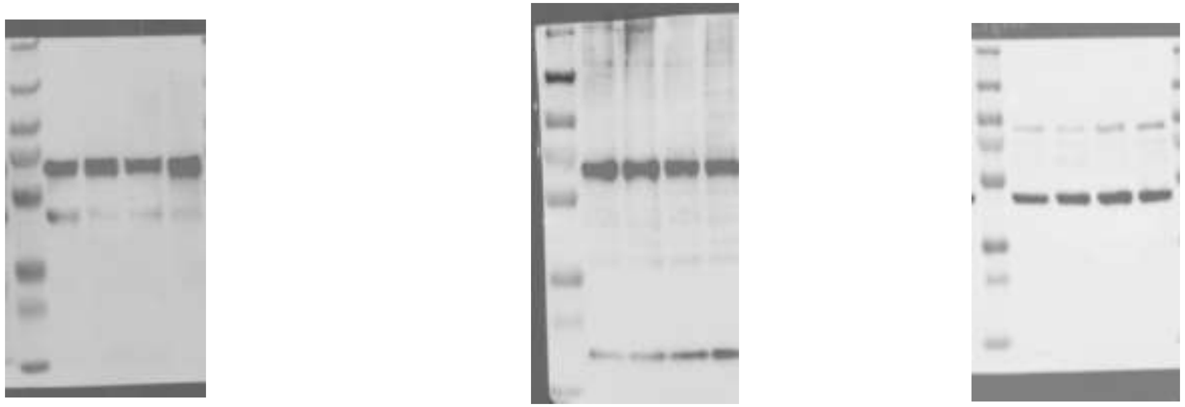

p-AKT (60 KDa) **AKT (56 KDa)** **β-actin (42 KDa)**

Control H/R DMSO-MSCs ATRA-MSCs

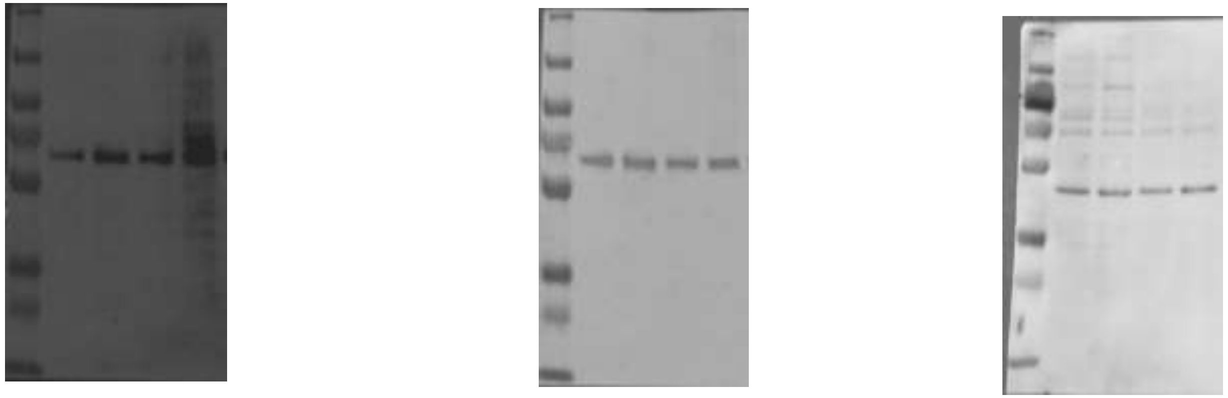

p-AKT (60 KDa) **AKT (56 KDa)** **β-actin (42 KDa)**

Figure 5

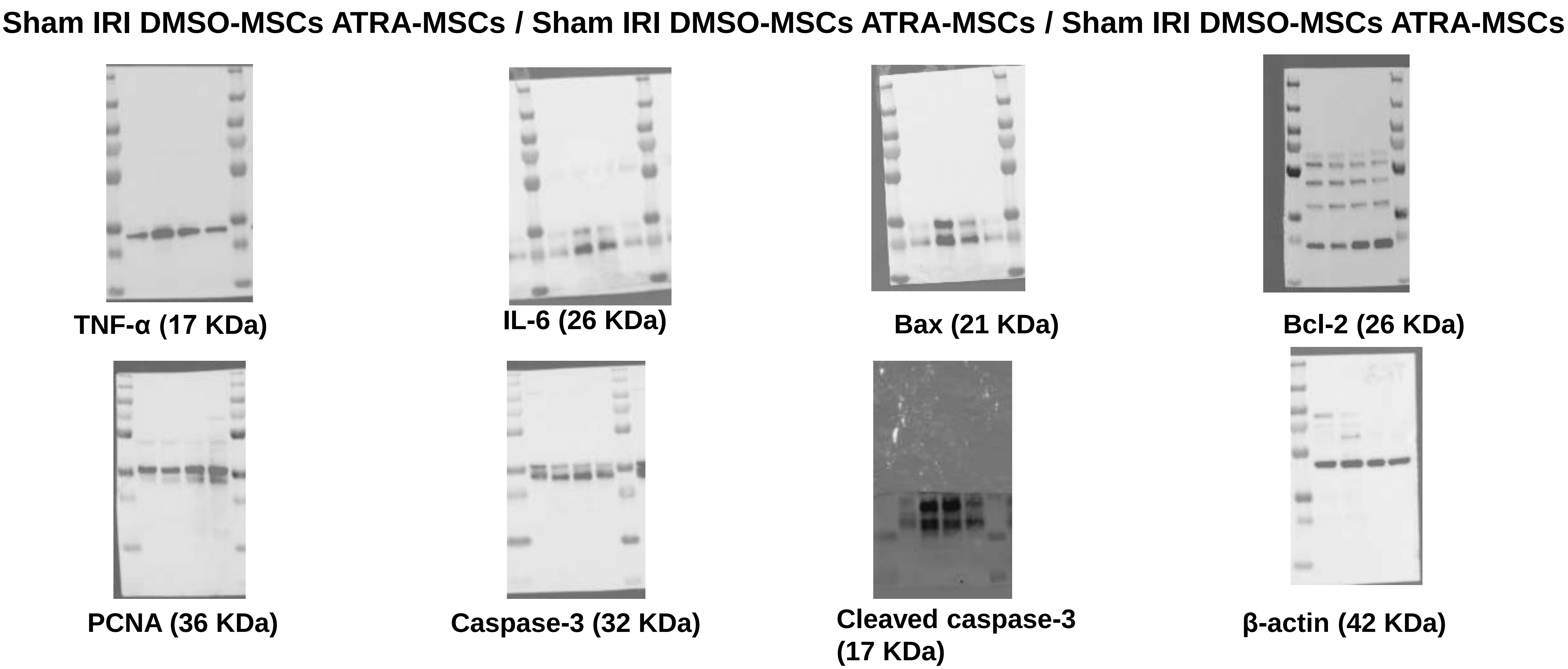
Sham IRI DMSO-MSCs ATRA-MSCs / Sham IRI DMSO-MSCs ATRA-MSCs / Sham IRI DMSO-MSCs ATRA-MSCs
TNF-α (17 KDa)
IL-6 (26 KDa)
Bax (21 KDa)
Bcl-2 (26 KDa)
PCNA (36 KDa)
Caspase-3 (32 KDa)
Cleaved caspase-3 (17 KDa)
β-actin (42 KDa)

Figure 6

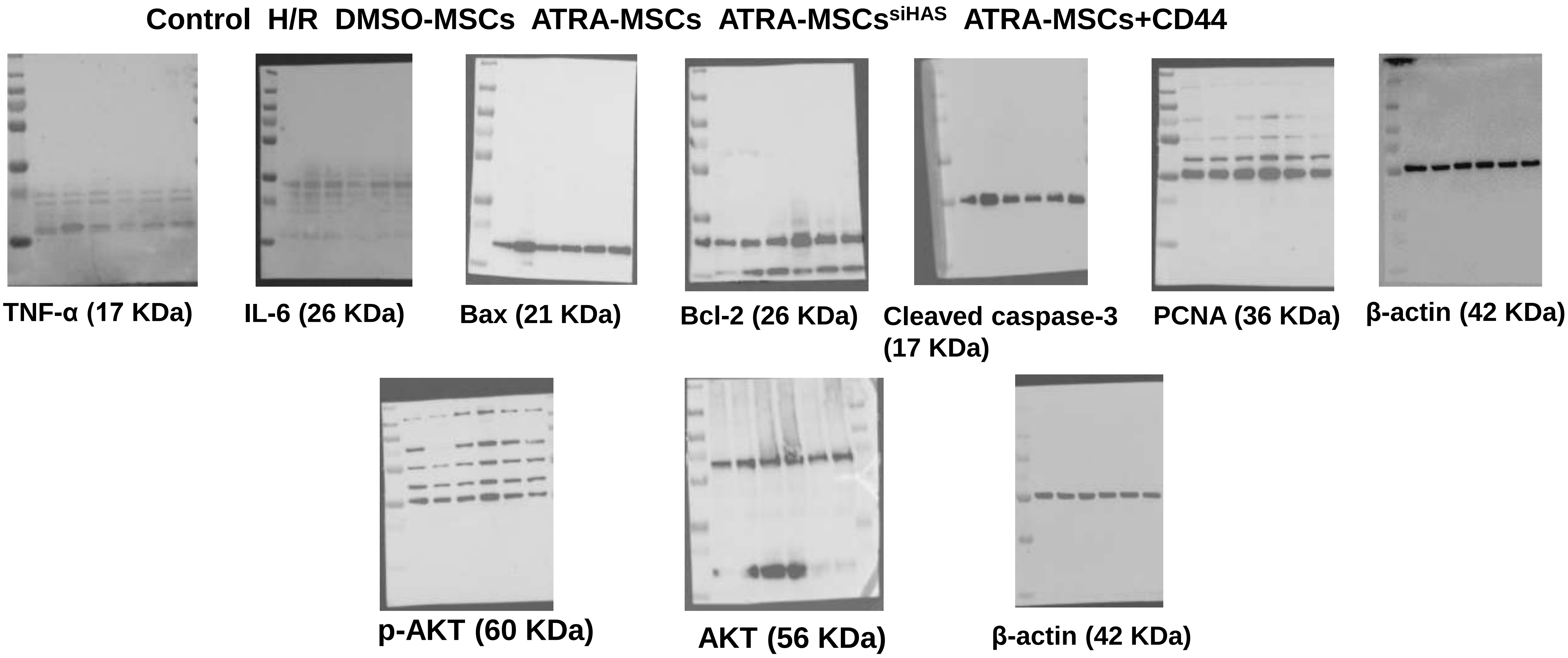
Control H/R DMSO-MSCs ATRA-MSCs ATRA-MSCs^{siHAS} ATRA-MSCs+CD44
TNF-α (17 KDa)
IL-6 (26 KDa)
Bax (21 KDa)
Bcl-2 (26 KDa)
Cleaved caspase-3 (17 KDa)
PCNA (36 KDa)
β-actin (42 KDa)
p-AKT (60 KDa)
AKT (56 KDa)
β-actin (42 KDa)
